# Novel Endo-β-*N*-Acetylglucosaminidases Derived from Human Fecal Samples Selectively Release *N*-Glycans from Model Glycoproteins

**DOI:** 10.3390/foods14081288

**Published:** 2025-04-08

**Authors:** Matthew Bolino, Nadini Haththotuwe Gamage, Hatice Duman, Odunayo Abiodun, Amilton S. De Mello, Sercan Karav, Steven A. Frese

**Affiliations:** 1 Department of Nutrition, University of Nevada, Reno, Reno, NV 89557, USA; 2 Department of Agriculture, Veterinary, and Rangeland Sciences, University of Nevada, Reno, Reno, NV 89557, USA; 3 Department of Molecular Biology and Genetics, Çanakkale Onsekiz Mart University, 17020 Çanakkale, Türkiye; 4 University of Nevada, Reno School of Medicine, Reno, NV 89557, USA

**Keywords:** enzymes, gut microbiome, alternative protein, ENGase, *N*-glycans

## Abstract

Three novel endo-β-*N*-acetylglucosaminidases (AVUL01, BCAC01, and BFIN01) classified as members of the glucoside hydrolase (GH) family 18 were identified from human fecal samples and then cloned and characterized for their ability to hydrolyze two distinct classes of *N*-glycans. Endo-β-*N*-acetylglucosaminidases (ENGases) are known for the hydrolysis of chitin and the *N*,*N*′-diacetylchitobiose core of *N*-linked glycans, depending on the glycan architecture. *N*-glycans have shown bioactivity as substrates in the human gut microbiome for microbes that encode ENGases, thus demonstrating their ecological relevance in the gut. However, distinct types of *N*-glycan structures, for example, oligomannosidic or complex, have been shown to enrich different microbes within the human gut. Novel advances in food technology have commercialized animal-derived dietary proteins with oligomannosidic instead of traditionally complex *N*-glycans using precision fermentation. This indicates that there is an unmet need to identify the classes of *N*-glycans that gut-derived ENGases act upon to determine whether these novel proteins alter gut ecology. AVUL01, BCAC01, and BFIN01 all demonstrated activity on exclusively oligomannosidic *N*-glycans from RNase B and bovine lactoferrin; however, they failed to show activity on complex or α-1,3-core fucosylated high-mannose *N*-glycans derived from fetuin and horseradish peroxidase, respectively. These results suggest that α-1,3 core fucosylation and complex *N*-glycan architecture inhibit the activity of AVUL01, BCAC01, and BFIN01. Furthermore, BFIN01 performed significantly better than BCAC01, resulting in a greater amount of *N*-glycans, suggesting that certain ENGases may possess enhanced specificity and kinetics as an evolutionary strategy to compete for resources.

## 1. Introduction

The gut microbiome is a dynamic and dense ecosystem whose biotic and abiotic features vary widely between individuals [1]. Gut microbes encounter a wide array of distinct substrates from the host’s diet. In humans, plant polysaccharides and other complex carbohydrates are the most abundant substrates that remain undigested by the host and pass into the large intestine where they are subject to microbial fermentation, resulting in the production of beneficial metabolites such as short-chain fatty acids [2]. When dietary fiber is low or absent, the microbiota resorts to alternative substrates that are present but may be derived from the host [3]. A major source of alternative substrates in the absence of complex carbohydrates are proteins [4], including host-derived mucin glycoproteins [5] or peptides derived from dietary protein [6].

Dietary proteins common in the diet contain a diverse array of bioactive *N*-linked glycans [7] and these *N*-glycans can act as a carbon source for key host-associated gut microbes such as *Bifidobacterium longum* subsp. *infantis* [8] and *Bacteroides thetaiotaomicron* [9]. *N*-linked glycans are attached to proteins post-translationally through a process known as glycosylation. Briefly, *N*-linked glycosylation begins in the endoplasmic reticulum (ER) by the specific recognition of an Asn-X-Ser/Thr sequence on a newly synthesized polypeptide by an oligosaccharyltransferase [10]. After the attachment of the highly conserved preassembled *N*-glycan core (Glc_3_Man_9_GlcNAc; [11], the *N*-glycan undergoes further modification in the Golgi apparatus [12], resulting in hundreds to thousands of potential *N*-glycan structures in an organism-specific manner [13].

Prior to *N*-glycan fermentation by gut microbes, a wide variety of microbiota-harboring enzymes known as glycoside hydrolases (GHs) hydrolyze various glycosidic bonds that construct the *N*-glycan [9]. Endo-β-*N*-acetylglucosaminidases (ENGases) are one of the initial GH enzymes in *N*-glycan acquisition through the hydrolysis of the *N*-glycan’s N,N′-diacetylchitobiose core, thus removing it from the peptide backbone [14]. ENGases are classified into two classes of GH families, GH18 and GH85, and specifically target the glycosidic bond between two *N*-acetylglucosamine monosaccharides, such as those found in *N*-glycans and chitin [15]. Evidence suggests that microbes with ENGases encoded in their genomes have greater ecological fitness when *N*-glycans are present [16]. For example, *Bacteroides thetaiotaomicron* utilizes its extracellular ENGases to remove the *N*-glycan from the polypeptide backbone and then transports the *N*-glycan into the periplasm for further degradation [9]. However, ENGases across organisms do not all display similar activity. In many cases, ENGase activity depends on the *N*-glycan’s monosaccharide and linkage construction. *N*-glycans containing sialic acid are one example that inhibits some ENGase-possessing microbes [17] while others are unaffected [9].

Novel advances in food technology have commercialized synthetically produced dietary protein, such as bovine lactoferrin [18], using precision fermentation with strikingly similar structure and functional benefits to its traditional counterpart. However, a recent study comparing the *N*-glycomes of synthetically produced and traditionally derived whey proteins highlighted significant differences in *N*-glycan structures between these protein sources [19]. Furthermore, these differences in *N*-glycan structures significantly impacted microbiota compositions and enriched for different microbes, suggesting that substrate acquisition by microbes is dependent on their ENGase specificities or “preference” towards different types and structures of *N*-glycans. In this report, we demonstrate that three novel ENGase sequences, AVUL01, BCAC01, and BFIN01, derived from human fecal samples exhibit varying levels of ENGase activity on oligomannosidic, complex, and α-1,3 core-fucosylated *N*-glycans from RNase B, bovine lactoferrin (bLF), fetuin, and horseradish peroxidase (HRP), respectively.

## 2. Methods

### 2.1. Sample Origins

This study was conducted in Nevada (USA) and was overseen by the University of Nevada, Reno Institutional Review Board (IRB), under approval #1751022. Adult participants were recruited from email listservs, local flyers at businesses, radio, and television interviews, and other print and online media solicitations. All methods were carried out in accordance with the relevant guidelines and regulations outlined by the Declaration of Helsinki. Adult residents of Nevada interested in participating in the study were pre-screened using an online intake portal to self-affirm that they met all of the inclusion criteria and none of the exclusion criteria prior to being consented and enrolled in the study. Informed consent was obtained from adults (over 18 years of age) who self-identified that they met the study inclusion criteria. Study inclusion criteria included individuals (1) over 18 years of age, (2) with no known and untreated infection (e.g., SARS-CoV-2), (3) consuming an ad libitum diet without medical intervention, and (4) literate in English and/or Spanish. Individuals were excluded from the study if they (1) had an active untreated infection within the last two weeks (e.g., SARS-CoV-2); (2) had a recent diagnosis of a malarial or parasitic infection; (3) were unable to stool spontaneously or had medically altered GI function (e.g., an ileostomy or colostomy); (4) were receiving a medically prescribed diet; (5) were currently hospitalized, critically ill, or were unable to give informed consent. Participants agreed to complete surveys of dietary intake and lifestyle habits, as well as demographic information. Additionally, participants agreed to provide a fecal sample.

Participants were instructed to return fecal samples so they could be processed and stored at −80 °C within 4 h of defecation. An amount of ~100 mg of fecal sample was collected in a ZymoResearch DNA/RNA Shield and stored as directed for subsequent DNA extraction at −80 °C. DNA was then extracted using a ZymoBIOMICS DNA MiniPrep kit (ZymoResearch, Irvine, CA, USA) according to the manufacturer’s instructions, which included a homogenization step using a FastPrep 24 homogenizer (MP Biomedicals, Santa Ana, CA, USA).

### 2.2. Sequencing and Identification of Endo-β-N-Acetylglucosaminidases

A total of 18 samples that were subjected to whole genomic DNA were prepared by the University of Nevada, Reno Genome Center, using an Illumina DNA library preparation kit for sequencing on a NextSeq 2000 with 150 bp paired-end reads. Samples were selected at random from fecal samples binned into each of the three microbiome enterotypes, which we have described previously [20]. Sequencing data were demultiplexed, reads were quality controlled, and adapters were removed using bbduk [21]. Reads from each sample were assembled using metaSPADES [22] and the resulting assemblies were binned using metaBAT2 [23]. Binned assemblies were then annotated using Prokka [24] and CAZymes were identified using dbcan2 [25] to identify candidate ENGases as members of the GH18 CAZyme family (EC3.2.1.96). All predicted ENGases were then aligned using MUSCLE [26] and identical sequences were identified and de-replicated for further analysis. Taxonomic identification of the de-replicated protein sequences was then performed using blastx [27,28] against the NCBI database and comparing the highest-ranked matches. Metagenomic sequencing data were also processed using HUMAnN (v3.9) [29] and a custom CAZyme database to estimate the abundance of CAZyme families among the samples after mapping to the Uniref90 database bundled with HUMAnN.

### 2.3. Cloning and Production of Candidate Enzymes

De-replicated candidate sequences were then selected for cloning and expression. Primers were designed to amplify the predicted sequences, omitting transmembrane domains, signal peptides, or predicted anchor domains, if present. These primers included a complementary overhang enabling in-frame cloning into p15TV-L. Expression from this system results in an in-frame *N*-terminal histidine tag and a TEV-cleavage site to remove the histidine tag, under the regulation of a T7 promoter.

Candidate ENGases were then amplified using the primers outlined in Table S1. For all reactions, amplification was performed using 2 μL of DNA obtained from the original fecal sample subjected to sequencing, diluted 1:10 in 10 mM Tris Cl, pH 8.0. The reactions were carried out using Phusion High-Fidelity PCR Master Mix with HF buffer (New England Biolabs, Ipswich, MA, USA) and 1 μL of 10 μM of each primer for the respective reactions. Cycling conditions involved 30 s at 98 °C, followed by 30 cycles of 10 s at 98 °C, 10 s at 60 °C, and 60 s at 72 °C. Finally, a 10 m final extension at 72 °C was performed. The amplification of desired PCR products was confirmed in a 1% agarose gel, and the resulting amplicon was purified using a ZymoResearch Clean and Concentrator-5 kit (ZymoResearch, Irvine, CA, USA).

BseRI-digested p15TV-L and the purified gene of interest were assembled using NEBuilder HiFi DNA Assembly Master Mix (New England Biolabs, Ipswich, MA, USA) and then transformed into T7 Express High-Efficiency *E. coli* (New England Biolabs, Ipswich, MA, USA) following the manufacturer’s instructions and then serially diluted for plating on LB agar containing carbenicillin (100 μg/mL). After incubating at 37 °C overnight, colonies were selected at random and subcultured, then grown in LB media containing carbenicillin overnight at 37 °C, with shaking at 250 RPM. An aliquot of cells was subjected to plasmid extraction with a ZR Plasmid Miniprep Classic kit (ZymoResearch, Irvine, CA USA). Plasmid identity was confirmed by restriction digestion against the predicted plasmid sequence. One clone with the correct plasmid sequence for each protein of interest was stored at −80 °C in 15% glycerol, and the remaining intact plasmid DNA was stored at −20 °C.

Cell lines were cultured in 5 mL of LB broth containing carbenicillin (100 μg/mL) and grown aerobically overnight at 37 °C in a shaking incubator at 250RPM. After 16 h, 3 volumes of Terrific Broth with added lactose (7.6 g/L) were added and shaking was increased (300RPM) with a reduction in temperature (24 °C). After 24 h of expansion at 24 °C, cells were harvested by centrifugation and lysing using BugBuster (Novagen) lysis buffer with 100 × protease inhibitor (CephamLifeSciences, Fulton, MD, USA). Lysate was then purified using the HisPur^TM^ Ni-NTA Purification kit (Thermo Scientific, Waltham, MA, USA) according to the manufacturer’s instructions. Raw cell lysate and the purified protein were examined using SDS-PAGE against the predicted size of the protein of interest. Confirmed protein then underwent buffer exchange to remove residual imidazole and was stored at standardized concentrations in 50% glycerol at −20 °C.

### 2.4. Comparison of N-Glycan Release by ENGases

The release of N-Glycan from AVUL01, BCAC01, and BFIN01 was performed using bLF (Sigma Aldrich, St. Louis, MO, USA), fetuin (Santa Ana, CA, USA), and HRP (Sigma Aldrich, St. Louis, MO, USA) as substrates using the previously described methodology [30]. Briefly, the total reaction volume (30 μL) consisted of 10 µL of enzyme (500 ng/µL in 50% glycerol), 3 µL of 200 mM sodium phosphate buffer (pH 5), 2 µL of deionized water, and 15 µL of denatured (95 °C for 5 min) bovine lactoferrin (10 mg/mL). Controls included substrate without enzymes. The same protocol was followed for HRP and fetuin (10 mg/mL) as well. Reactions were performed in quadruplets for each enzyme at a temperature of 37 °C. The reaction mixtures were incubated for 16 h at 37 °C and the reaction ended and the release of N-glycans was quantified by precipitating protein with 3-volumes of ice-cold ethanol. The plates were then incubated at −20 °C for one hour or more, followed by centrifuging the samples, recovering the supernatant, and quantifying the residual glycans after drying to remove the ethanol, using a standard plate-based phenol sulfuric assay [31].

### 2.5. Statistical Analyses

Statistical tests were performed in R (v. 4.2.2) [32]. Protein abundances were compared between groups using a nonparametric Kruskal–Wallis test [33] and a nonparametric Wilcoxon test [34] using *ggpubr* (v. 0.4.0) and *rstatix* (v. 0.7.0) R packages [35,36].

## 3. Results

### 3.1. Identification and Production of Novel ENGases from Human Fecal Samples

**Figure 1:**
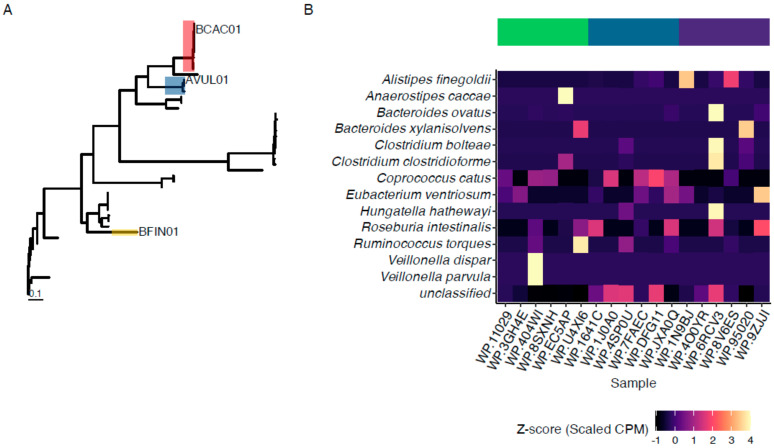
*Protein sequence alignment and abundance of predicted GH18 proteins*. (**A**) Protein sequences identified as GH18 were extracted and aligned. Three sequences were selected for comparison (BCAC01, AVUL01, and BFIN01) as representatives of de-replicated sequences (red, blue, or yellow shaded leaves, respectively). (**B**) The abundances of GH18-annotated proteins were also estimated across the bacterial species in the samples examined in this study, with the six samples from each enterotype (E1, E2, and E3 respectively, as indicated by the green, blue, or purple bar at the top) and the taxa with mapped predicted GH18-family proteins indicated by the Z-scaled counts per million mapped reads (Z-scaled CPM).

After assembly and annotation, sequences identified as candidate ENGases were aligned, and fourteen distinct sequences were identified from the dataset. After collapsing identical sequences, the centermost representative sequences were considered for characterization (Figure 1A). Then, having analyzed the sequences, we identified three sequences with likely ENGase activity from closely related sequences (AVUL01 and BCAC01) and a third, more distant sequence (BFIN01) and selected these three enzymes for cloning and expression after removing cell membrane anchor domains expected to limit protein expression in vitro.

When comparing the translated amino acid sequences, AVUL01 was predicted to originate from *Alistipes onderdonkii* subsp. *vulgaris*, a member of the *Rikenellaceae* family and order *Bacteroidales*. The closest amino acid sequence match to AVUL01 that has been functionally characterized originates from *Bacteroides thetaiotaomicron*, but that protein is less than 63% similar, ref [37] suggesting that while these two protein sequences may have some functional homology, there is no evidence that they have matching enzymatic characteristics. BCAC01 is predicted to originate from *Bacteroides caccae, B. ovatus*, or *B. xylanisolvens*, all members of the *Bacteroidaceae* family. The closest predicted amino acid sequences are also found among *B. ovatus* and *B. xylanisolvens*, but these proteins have not yet been functionally characterized. Finally, BFIN01 was found to have significant sequence homology with a nucleotide sequence in the *Alistipes* (formerly *Bacteroides*) *finegoldii* genome only, with no other significant sequence matches. When comparing the predicted amino acid sequence to the NCBI nr database, there were only significant matches within the *A. finegoldii* and *B. stercoris* genomes, with all other results bearing less than 75% sequence homology, indicating that the characteristics of this predicted protein sequence are likely to be significantly different from even other homologs across *Bacteroides*.

To identify the abundance of GH18-family enzymes across the input sample, we compared the abundance across species identified using HUMAnN (see Methods). As we selected six random samples for sequencing from each of three microbiome enterotypes (E1, E2, and E3), we examined the abundance of GH18 family CAZymes and found that there were no significant differences (*p* > 0.05) between enterotypes in terms of the abundance of proteins with predicted membership in the GH18 family. We also noted that the prevalence of these species-specific predicted GH18 family enzymes was highly variable across individuals, and per-species comparisons between these predicted GH18 family enzymes were also not significant (*p* > 0.05). However, the overall abundance of these enzymes also did not differ between enterotypes (*p* > 0.05), with a mean of 1.85 ± 2.17 SD, 3.64 ± 2.85 SD, and 6.67 ± 11.1 SD for E1, E2, and E3 samples, respectively. While the mean abundance of these groups may differ, the elevated mean in E3 was a result of one sample with an elevated abundance of a GH18 among *B. ovatus* in that community (Figure 1B).

### 3.2. ENGases Display Catalytic Activity Exclusively on Oligomannose Glycoproteins

**Figure 2:**
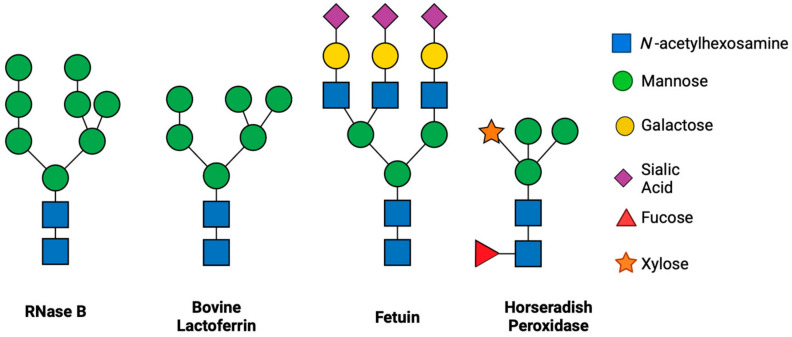
*Schematic representation of the most abundant N-glycan structures across glycoproteins.* Structure representation of the most abundant *N*-glycan on RNase B [38], bovine lactoferrin [39], fetuin [40], and horseradish peroxidase [41].

After cloning and expression in *E. coli*, followed by purification using a Ni-resin column (see Methods, Supplementary Figure S1), we quantified the amount of enzyme produced on a Qubit 4 Fluorometer (ThermoFisher Scientific; Waltham, MA, USA). After purification and concentration, AVUL01, BCAC01, and BFIN01 produced approximately 314, 232, and 326 mg of enzyme per liter. We then compared the ability of these novel enzymes (AVUL01, BCAC01, and BFIN01) to remove oligomannosidic, complex, or core fucosylated *N*-glycans (Figure 2) from the four glycoprotein sources RNase B, bLF, fetuin, and HRP.

**Figure 3:**
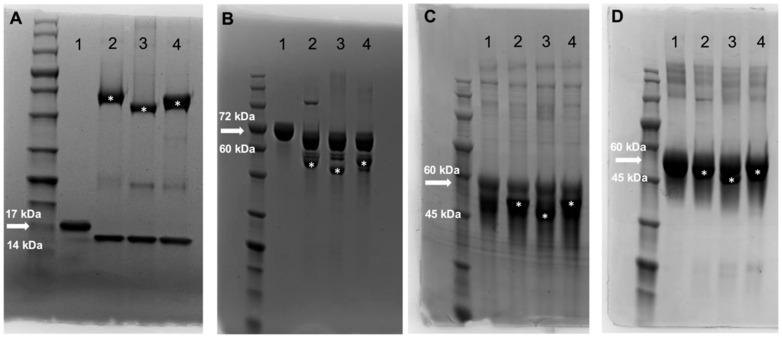
*Enzymatic deglycosylation by ENGases using SDS-PAGE Coomassie staining*: (**A**) 4–20% SDS-PAGE gel staining for RNase B alone (1) or treated with AVUL01 (2), BCAC01 (3), or BFIN (4), indicating activity represented by a gel shift; 10% SDS-PAGE gel staining for (**B**) bovine lactoferrin, (**C**) fetuin, or (**D**) HRP alone (1) or treated with AVUL01 (2), BCAC01 (3), or BFIN (4), indicating activity represented by a gel shift. Arrows indicate intact, glycosylated proteins. Asterisks represent enzymes stained in the gels.

RNase B contains one oligomannosidic *N*-glycan and was used as an initial test for ENGase activity. RNase B is 17 kDa when the *N*-glycan is bound [38] and ~14 kDa when the *N*-glycan is cleaved [30], resulting in a gel shift when visualized using SDS-PAGE. Enzyme concentrations were standardized to 500 ng per μL and reactions were carried out at a pH of 5 and 37 °C. After 16 h of incubation, all three enzymes displayed clear ENGase activity when visualized by SDS-PAGE with Coomassie stain. The gel indicates a size shift compared to the no-enzyme control (Figure 3A). We then carried out the same reactions using bovine lactoferrin, fetuin, and HRP to test the enzymatic activity of AVUL01, BCAC01, and BFIN01 on different types of *N*-glycans prior to quantifying the concentration of *N*-glycan release. Similar to the RNase B gel, all three enzymes resulted in a visible lactoferrin gel shift, signifying the release of one or more of its *N*-glycans (Figure 3B). However, fetuin and HRP did not result in a gel shift (Figure 3C,D), suggesting that all three enzymes are not active on complex or α-1,3-core fucosylated *N*-glycans.

**Figure 4:**
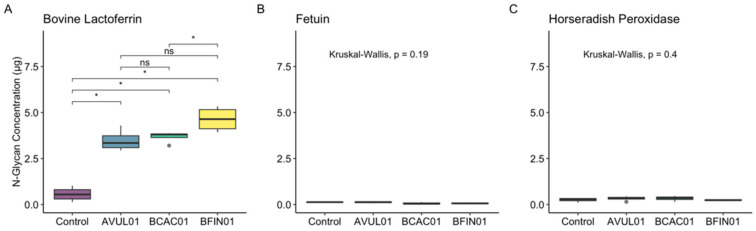
*Comparison of released N-glycan concentrations across enzymes.* Boxplots comparing the amount (μg total) of released *N*-glycans across enzymes on (**A**) bovine lactoferrin, (**B**) fetuin, and (**C**) horseradish peroxidase. ns signifies not significant, and an asterisk denotes a *p* < 0.05.

In addition to SDS-PAGE Coomassie staining to test for enzyme activity, additional reactions under the same conditions of pH 5 and 37 °C were performed with each glycoprotein to quantify the release of *N*-glycans across enzymes as a proxy for efficiency. To quantify the concentration of released *N*-glycans, phenol sulfuric assays were performed, and the OD of each sample was measured at 485 nm. All enzymes released a significantly greater quantity of *N*-glycans from bovine lactoferrin compared to the no-enzyme control (*p* < 0.05, Figure 4A), signifying that these enzymes show catalytic activity on lactoferrin *N*-glycan structures. No significant differences in released *N*-glycan concentration were detected between AVUL01 and both BCAC01 and BFIN01 (*p* > 0.05); however, the concentration of released *N*-glycans by BFIN01 was significantly greater than that of BCAC01 (*p* < 0.05) measured by a nonparametric Wilcoxon test (Figure 4A). However, since bovine lactoferrin contains a mixture of complex and oligomannosidic structures, additional reactions were performed with fetuin, which contains exclusively complex *N*-glycans. All three enzymes failed to release *N*-glycans from fetuin compared to the no-enzyme control (Figure 4B), suggesting that these enzymes are only active on oligomannosidic structures.

Finally, reactions with HRP were performed to identify whether these enzymes were restricted by monosaccharide modifications that are commonly known for inhibiting the catalytic activity of other glycosidases, such as α-1,3 core-fucosylation [42], shown in Figure 2. Interestingly, α-1,3 core-fucosylation inhibited the catalytic activity of all three enzymes, as no significant differences in released *N*-glycans were detected compared to the no-enzyme control (*p* > 0.05, Figure 4C).

## 4. Discussion

In this study, we used human fecal samples to identify and characterize the catalytic activity of three novel ENGases for the release of three *N*-glycan types found on RNase B, bovine lactoferrin, fetuin, and horseradish peroxidase. These *N*-glycans are protein-bound carbohydrates similar to fibers, and microbes with the ability to utilize *N*-glycans as a substrate demonstrate enhanced fitness [16,43] when fiber is low or absent. In most cases, ENGase activity is the microbe’s initial step towards substrate acquisition by removing the *N*-glycan from the peptide backbone prior to transport, deconstruction, and metabolism [8].

As it relates to *N*-glycans, not all ENGases display the same specificities or preferences. For example, Endo-H derived from *Streptomyces plicatus* shows activity on oligomannosidic and hybrid but not complex *N*-glycans [44]. In a more extreme example of narrow ENGase specificity, Endo-S derived from *Streptococcus pyogenes* displays activity on complex *N*-glycans only from human IgG glycoforms but no other glycoproteins [45]. This wide range of ENGase specificities across microbes likely indicates microbe adaptations to specific but frequently present *N*-glycans within a given environment.

Novel food biotechnology has advanced significantly over the last decade and has commercialized dietary protein production, such as bovine whey and lactoferrin, using precision fermentation [18,19], resulting in proteins with oligomannosidic instead of traditional complex *N*-glycans. Importantly, Bolino et al. recently highlighted that differences in *N*-glycans, such as oligomannosidic vs complex, result in the enrichment of different microbes in vitro [19], supporting ENGase specificity as an important consideration for microbiome modulation. Here, we demonstrated the catalytic activity of three novel ENGases, AVUL01, BCAC01, and BFIN01, on exclusively oligomannosidic structures, but not complex or α-1,3 core-fucosylated *N*-glycans. While we did not identify the released *N*-glycan structures from the bovine lactoferrin, it is likely that they were exclusively oligomannosidic, according to the lack of catalytic activity on complex structures demonstrated on fetuin. We also found significant differences in *N*-glycan concentration between BFIN01 and BCAC01, suggesting possible evolutionary adaptations of each enzyme-possessing microbe to ecological niches with distinct conditions.

Additionally, the predicted microbes in which AVUL01, BCAC01, and BFIN01 were derived from, *Alistipes onderdonkii* subsp. *vulgaris*, *Bacteroides caccae*, and *Alistipes finegoldii*, respectively, have all been previously identified to encode for at least 10 proteins each that contain a Domain of Unknown Function 1735 (DUF1735) associated with carbohydrate degradation [46]. The only previously investigated DUF1735-containing protein found in *Bacteroides thetaiotaomicron* that contained a C-terminal LamG3 domain, which was the most frequent and representative domain architecture for DUF1735-containing proteins across bacteria [46], was located within a polysaccharide utilization loci (PUL72) which is involved in oligomannosidic *N*-glycan degradation [47], supporting the results mentioned above.

While we characterized the catalytic activity of these novel enzymes on different *N*-glycan classes, one limitation of this study is we did not characterize the released *N*-glycan structures, which may have provided additional insights as to whether certain reaction conditions release distinct sets, as demonstrated by [48]. It is also important to note that we isolated the enzyme from the organism to test enzyme activity directly; however, it is possible and likely that these enzymes perform differently when expressed within the organism in a complex ecosystem, like the human gut microbiota [49].

In conclusion, three novel ENGases in the GH18 family were mined from human fecal samples belonging to phylogenetically different organisms. These ENGases demonstrated catalytic activity on exclusively oligomannosidic but not complex *N*-glycan types and were inhibited by α-1,3 core-fucosylation. Although catalytic activity was determined across distinct substrates, further studies are needed to characterize the enzyme kinetics and released *N*-glycan structures to gain a better understanding of studying bioactive *N*-glycans.

## Data Availability

Sequencing data generated in this study are available at the NCBI under BioProject PRJNA1219209. Sequences for the enzymes described in this study are available under accession numbers PV240270-PV240272 in the NCBI GenBank database.

## Supplementary Materials

The following supporting information can be downloaded at: https://www.mdpi.com/article/10.3390/foods14081288/s1, Table S1: Primer sequences used in this study; Figure S1: 10% SDS-PAGE gel stained with Coomassie blue before and after enzyme purification. A representative gel showing protein from *E. coli* containing AVUL01 plasmid prior to induction (1), after induction (2) and after purification (3). *E. coli* containing BCAC01 plasmid prior to induction (4), after induction (5) and after purification (6). *E. coli* containing BFIN01 plasmid prior to induction (7), after induction (8) and after purification (9).

## References

[1] Berg G., Rybakova D., Fischer D., Cernava T., Vergès M.-C.C., Charles T., Chen X., Cocolin L., Eversole K., Corral G.H. (2020). Microbiome Definition Re-Visited: Old Concepts and New Challenges. Microbiome.

[2] Holscher H.D. (2017). Dietary Fiber and Prebiotics and the Gastrointestinal Microbiota. Gut Microbes.

[3] Koropatkin N.M., Cameron E.A., Martens E.C. (2012). How Glycan Metabolism Shapes the Human Gut Microbiota. Nat. Rev. Microbiol..

[4] Makki K., Deehan E.C., Walter J., Bäckhed F. (2018). The Impact of Dietary Fiber on Gut Microbiota in Host Health and Disease. Cell Host Microbe.

[5] Desai M.S., Seekatz A.M., Koropatkin N.M., Kamada N., Hickey C.A., Wolter M., Pudlo N.A., Kitamoto S., Terrapon N., Muller A. (2016). A Dietary Fiber-Deprived Gut Microbiota Degrades the Colonic Mucus Barrier and Enhances Pathogen Susceptibility. Cell.

[6] Windey K., De Preter V., Verbeke K. (2012). Relevance of Protein Fermentation to Gut Health. Mol. Nutr. Food Res..

[7] Bolino M., Avcı İ., Kayili H.M., Duman H., Salih B., Karav S., Frese S.A. (2025). Identification and Comparison of N-Glycome Profiles from Common Dietary Protein Sources. Food Chem. X.

[8] Karav S., Le Parc A., Leite Nobrega de Moura Bell J.M., Frese S.A., Kirmiz N., Block D.E., Barile D., Mills D.A. (2016). Oligosaccharides Released from Milk Glycoproteins Are Selective Growth Substrates for Infant-Associated Bifidobacteria. Appl. Environ. Microbiol..

[9] Briliūtė J., Urbanowicz P.A., Luis A.S., Baslé A., Paterson N., Rebello O., Hendel J., Ndeh D.A., Lowe E.C., Martens E.C. (2019). Complex N-Glycan Breakdown by Gut Bacteroides Involves an Extensive Enzymatic Apparatus Encoded by Multiple Co-Regulated Genetic Loci. Nat. Microbiol..

[10] Breitling J., Aebi M. (2013). N-Linked Protein Glycosylation in the Endoplasmic Reticulum. Cold Spring Harb. Perspect. Biol..

[11] Aebi M. (2013). N-Linked Protein Glycosylation in the ER. Biochim. Biophys. Acta (BBA)-Mol. Cell Res..

[12] Wang P., Wang H., Gai J., Tian X., Zhang X., Lv Y., Jian Y. (2017). Evolution of Protein N-Glycosylation Process in Golgi Apparatus Which Shapes Diversity of Protein N-Glycan Structures in Plants, Animals and Fungi. Sci. Rep..

[13] Gagneux P., Panin V., Hennet T., Aebi M., Varki A., Varki A., Cummings R.D., Esko J.D., Stanley P., Hart G.W., Aebi M., Mohnen D., Kinoshita T., Packer N.H., Prestegard J.H. (2022). Evolution of Glycan Diversity. Essentials of Glycobiology.

[14] Duman H., Kaplan M., Arslan A., Sahutoglu A.S., Kayili H.M., Frese S.A., Karav S. (2021). Potential Applications of Endo-β-N-Acetylglucosaminidases from *Bifidobacterium longum* Subspecies Infantis in Designing Value-Added, Next-Generation Infant Formulas. Front. Nutr..

[15] Funkhouser J.D., Aronson N.N. (2007). Chitinase Family GH18: Evolutionary Insights from the Genomic History of a Diverse Protein Family. BMC Evol. Biol..

[16] Barratt M.J., Nuzhat S., Ahsan K., Frese S.A., Arzamasov A.A., Sarker S.A., Islam M.M., Palit P., Islam M.R., Hibberd M.C. (2022). *Bifidobacterium infantis* Treatment Promotes Weight Gain in Bangladeshi Infants with Severe Acute Malnutrition. Sci. Transl. Med..

[17] Garbe J., Sjögren J., Cosgrave E.F.J., Struwe W.B., Bober M., Olin A.I., Rudd P.M., Collin M. (2014). EndoE from *Enterococcus faecalis* Hydrolyzes the Glycans of the Biofilm Inhibiting Protein Lactoferrin and Mediates Growth. PLoS ONE.

[18] Skoog E.C., Castagna V.F., Omer S., Madigan J., Flagg V., Burrick K., Jiang R., Du X., Lönnerdal B., Schnitzler A. (2025). Structure and Function of Fermentation-Derived Bovine Lactoferrin Produced from *Komagataella phaffii*. Biochem. Cell Biol..

[19] Bolino M., Duman H., Avcı İ., Kayili H.M., Petereit J., Zundel C., Salih B., Karav S., Frese S.A. (2024). Proteomic and N-Glycomic Comparison of Synthetic and Bovine Whey Proteins and Their Effect on Human Gut Microbiomes. bioRxiv.

[20] Flores Martinez K.E., Bloszies C.S., Bolino M.J., Henrick B.M., Frese S.A. (2024). Hemp Hull Fiber and Two Constituent Compounds, N-Trans-Caffeoyltyramine and N-Trans-Feruloyltyramine, Shape the Human Gut Microbiome in Vitro. Food Chem. X.

[21] Bushnell B., Rood J., Singer E. (2017). BBMerge–Accurate Paired Shotgun Read Merging via Overlap. PLoS ONE.

[22] Nurk S., Meleshko D., Korobeynikov A., Pevzner P.A. (2017). metaSPAdes: A New Versatile Metagenomic Assembler. Genome Res..

[23] Kang D.D., Li F., Kirton E., Thomas A., Egan R., An H., Wang Z. (2019). MetaBAT 2: An Adaptive Binning Algorithm for Robust and Efficient Genome Reconstruction from Metagenome Assemblies. PeerJ.

[24] Seemann T. (2014). Prokka: Rapid Prokaryotic Genome Annotation. Bioinformatics.

[25] Huang L., Zhang H., Wu P., Entwistle S., Li X., Yohe T., Yi H., Yang Z., Yin Y. (2018). dbCAN-Seq: A Database of Carbohydrate-Active Enzyme (CAZyme) Sequence and Annotation. Nucleic Acids Res..

[26] Edgar R.C. (2004). MUSCLE: Multiple Sequence Alignment with High Accuracy and High Throughput. Nucleic Acids Res..

[27] Camacho C., Coulouris G., Avagyan V., Ma N., Papadopoulos J., Bealer K., Madden T.L. (2009). BLAST+: Architecture and Applications. BMC Bioinform..

[28] Altschul S.F., Gish W., Miller W., Myers E.W., Lipman D.J. (1990). Basic Local Alignment Search Tool. J. Mol. Biol..

[29] Beghini F., McIver L.J., Blanco-Míguez A., Dubois L., Asnicar F., Maharjan S., Mailyan A., Manghi P., Scholz M., Thomas A.M. (2021). Integrating Taxonomic, Functional, and Strain-Level Profiling of Diverse Microbial Communities with bioBakery 3. eLife.

[30] Karav S., Bell J.M.L.N.D.M., Le Parc A., Liu Y., Mills D.A., Block D.E., Barile D. (2015). Characterizing the Release of Bioactive N-Glycans from Dairy Products by a Novel Endo-β-N-Acetylglucosaminidase. Biotechnol. Prog..

[31] Masuko T., Minami A., Iwasaki N., Majima T., Nishimura S.-I., Lee Y.C. (2005). Carbohydrate Analysis by a Phenol-Sulfuric Acid Method in Microplate Format. Anal. Biochem..

[32] R Core Team (2022). R: A Language and Environment for Statistical Computing.

[33] Kruskal W.H., Wallis W.A. (1952). Use of Ranks in One-Criterion Variance Analysis. J. Am. Stat. Assoc..

[34] Wilcoxon F., Kotz S., Johnson N.L. (1992). Individual Comparisons by Ranking Methods. Breakthroughs in Statistics: Methodology and Distribution.

[35] Kassambara A. (2022). Ggpubr: “ggplot2” Based Publication Ready Plots. https://rpkgs.datanovia.com/ggpubr/.

[36] Kassambara A. (2022). Rstatix: Pipe-Friendly Framework for Basic Statistical Tests. https://rdrr.io/cran/rstatix/.

[37] Trastoy B., Du J.J., Klontz E.H., Li C., Cifuente J.O., Wang L.-X., Sundberg E.J., Guerin M.E. (2020). Structural Basis of Mammalian High-Mannose N-Glycan Processing by Human Gut Bacteroides. Nat. Commun..

[38] Prien J.M., Ashline D.J., Lapadula A.J., Zhang H., Reinhold V.N. (2009). The High Mannose Glycans from Bovine Ribonuclease B Isomer Characterization by Ion Trap MS. J. Am. Soc. Mass Spectrom..

[39] Gnanesh Kumar B.S., Mattad S. (2021). Comprehensive Analysis of Lactoferrin N-Glycans with Site-Specificity from Bovine Colostrum Using Specific Proteases and RP-UHPLC-MS/MS. Int. Dairy J..

[40] Marie A.-L., Ray S., Ivanov A.R. (2023). Highly-Sensitive Label-Free Deep Profiling of N-Glycans Released from Biomedically-Relevant Samples. Nat. Commun..

[41] Kurosaka A., Yano A., Itoh N., Kuroda Y., Nakagawa T., Kawasaki T. (1991). The Structure of a Neural Specific Carbohydrate Epitope of Horseradish Peroxidase Recognized by Anti-Horseradish Peroxidase Antiserum. J. Biol. Chem..

[42] Wang T., Cai Z.P., Gu X.Q., Ma H.Y., Du Y.M., Huang K., Voglmeir J., Liu L. (2014). Discovery and Characterization of a Novel Extremely Acidic Bacterial N-Glycanase with Combined Advantages of PNGase F and A. Biosci. Rep..

[43] Crouch L.I., Urbanowicz P.A., Baslé A., Cai Z.-P., Liu L., Voglmeir J., Melo Diaz J.M., Benedict S.T., Spencer D.I.R., Bolam D.N. (2022). Plant N-Glycan Breakdown by Human Gut Bacteroides. Proc. Natl. Acad. Sci. USA.

[44] Freeze H.H., Kranz C. (2010). Endoglycosidase and Glycoamidase Release of N-Linked Glycans. Curr. Protoc. Mol. Biol..

[45] Trastoy B., Klontz E., Orwenyo J., Marina A., Wang L.-X., Sundberg E.J., Guerin M.E. (2018). Structural Basis for the Recognition of Complex-Type N-Glycans by Endoglycosidase S. Nat. Commun..

[46] Hameleers L., Gaenssle L.A., Bertran-Llorens S., Pijning T., Jurak E. (2024). Polysaccharide Utilization Loci Encoded DUF1735 Likely Functions as Membrane-Bound Spacer for Carbohydrate Active Enzymes. FEBS Open Bio.

[47] Cuskin F., Lowe E.C., Temple M.J., Zhu Y., Cameron E.A., Pudlo N.A., Porter N.T., Urs K., Thompson A.J., Cartmell A. (2015). Human Gut Bacteroidetes Can Utilize Yeast Mannan through a Selfish Mechanism. Nature.

[48] Karav S., Kuddus M. (2019). Application of a Novel Endo-β-N-Acetylglucosaminidase to Isolate an Entirely New Class of Bioactive Compounds: *N*-Glycans. Enzymes in Food Biotechnology.

[49] Martens E.C., Kelly A.G., Tauzin A.S., Brumer H. (2014). The Devil Lies in the Details: How Variations in Polysaccharide Fine-Structure Impact the Physiology and Evolution of Gut Microbes. J. Mol. Biol..

