# Supplementary figures and images for: Novel Endo-β-N-Acetylglucosaminidases Derived from Human Fecal Samples Selectively Release N-Glycans from Model Glycoproteins
Source: Foods. 2025 Apr 8;14(8):1288. doi: 10.3390/foods14081288 (PMC12025955; doi:10.3390/foods14081288)

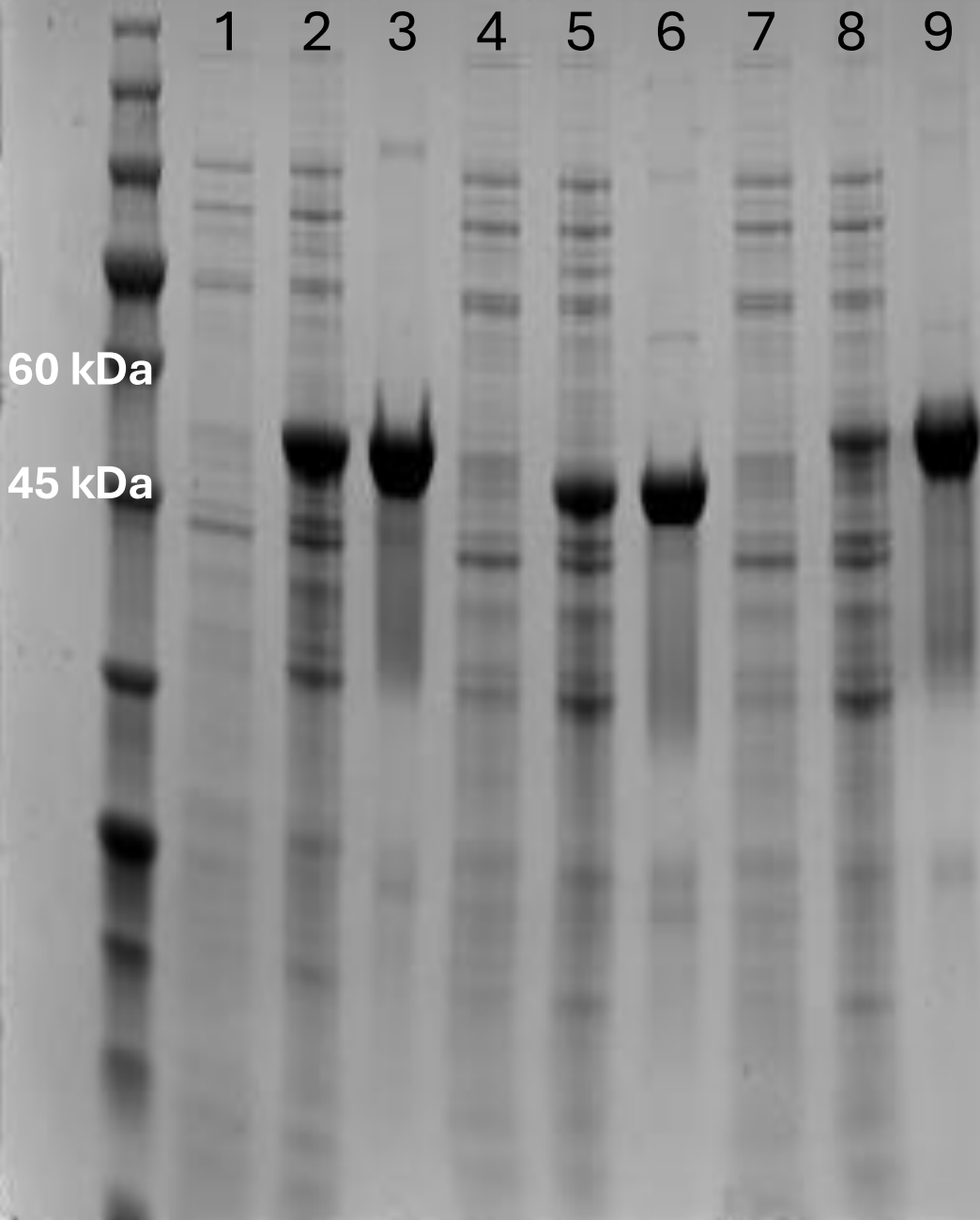

Supplement: Supplementary file 1 [file foods-14-01288-s001.zip › Supp Figure S1.pdf]
